# Supplementary material for: Myelin Disruption, Neuroinflammation, and Oxidative Stress Induced by Sulfite in the Striatum of Rats Are Mitigated by the pan-PPAR agonist Bezafibrate
Source: Cells. 2023 Jun 6;12(12):1557. doi: 10.3390/cells12121557 (PMC10296939; doi:10.3390/cells12121557)
Supplement: Supplementary file 1 [file cells-12-01557-s001.zip › cells-2301116-supplementary.pdf]

cells MDPI

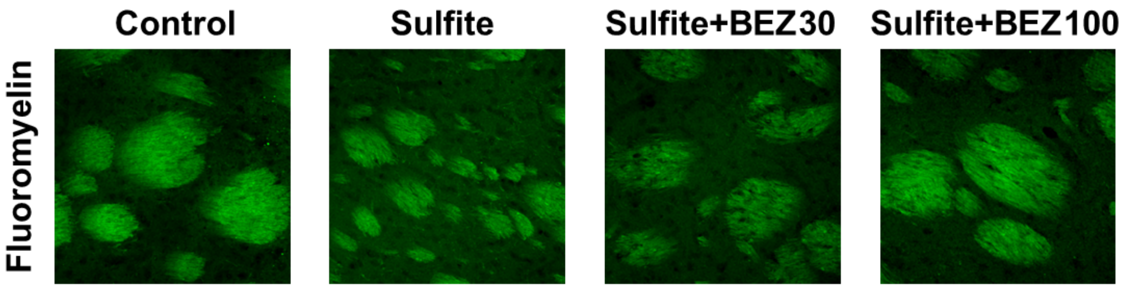

Supplementary Figure S1. Bezafibrate (BEZ) post-treatment mitigates sulfite-induced reduction in Fluoromyelin staining in rat striatum 7 days after sulfite injection (2 μmol). Animals were post-treated with BEZ (30 or 100 mg/kg/day) for 7 days after sulfite infusion. Representative images are shown.

Supplementary Table S1. Genes analyzed by quantitative RT-PCR.

Gene	Assay ID
Interleukin-6 (IL-6)	#Rn01410330_m1
Interleukin-1β (IL-1β)	#Rn00580432_m1
Interleukin-10 (IL-10)	#Rn00563409_m1
Tumor necrosis factor-α (TNF-α)	#Rn99999017_m1
IL1 receptor type I (IL1R1)	#Rn00565482_m1
TNF receptor 1 (TNFR1)	#Rn01492348_m1
Nuclear factor kappa B p65 subunit (NFκB p65)	#Rn01502266_m1
Cyclooxygenase-2 (COX-2)	#Rn01483828_m1
Inducible nitric oxide synthase (iNOS)	#Rn00561646_m1
Nuclear factor erythroid 2-related factor 2 (Nrf2)	#Rn00582415_m1
Heme oxygenase-1 (HO-1)	#Rn01536933_ m1
Superoxide dismutase 1 (SOD1)	#Rn00566938_m1
Superoxide dismutase 2 (SOD2)	#Rn00690588_g1
β-actin	#Rn00667869_m1
